# A Comparative Study of Multiple Deep Learning Models Based on Multi-Input Resolution for Breast Ultrasound Images

**DOI:** 10.3389/fonc.2022.869421

**Published:** 2022-07-07

**Authors:** Huaiyu Wu, Xiuqin Ye, Yitao Jiang, Hongtian Tian, Keen Yang, Chen Cui, Siyuan Shi, Yan Liu, Sijing Huang, Jing Chen, Jinfeng Xu, Fajin Dong

**Affiliations:** ^1^ Department of Ultrasound, First Clinical College of Jinan University, Second Clinical College of Jinan University, First Affiliated Hospital of Southern University of Science and Technology, Shenzhen People’s Hospital, Shenzhen, China; ^2^ Research and Development Department, Microport Prophecy, Shanghai, China; ^3^ Research and Development Department, Illuminate Limited Liability Company, Shenzhen, China; ^4^ The Key Laboratory of Cardiovascular Remodeling and Function Research, Chinese Ministry of Education and Chinese Ministry of Health, and The State and Shandong Province Joint Key Laboratory of Translational Cardiovascular Medicine, Cheeloo College of Medicine, Shandong University, Qilu Hospital of Shandong University, Jinan, China

**Keywords:** breast cancer, deep learning, ultrasound, resolution, artifical intelligence

## Abstract

**Purpose:**

The purpose of this study was to explore the performance of different parameter combinations of deep learning (DL) models (Xception, DenseNet121, MobileNet, ResNet50 and EfficientNetB0) and input image resolutions (REZs) (224 × 224, 320 × 320 and 488 × 488 pixels) for breast cancer diagnosis.

**Methods:**

This multicenter study retrospectively studied gray-scale ultrasound breast images enrolled from two Chinese hospitals. The data are divided into training, validation, internal testing and external testing set. Three-hundreds images were randomly selected for the physician-AI comparison. The Wilcoxon test was used to compare the diagnose error of physicians and models under *P*=0.05 and 0.10 significance level. The specificity, sensitivity, accuracy, area under the curve (AUC) were used as primary evaluation metrics.

**Results:**

A total of 13,684 images of 3447 female patients are finally included. In external test the 224 and 320 REZ achieve the best performance in MobileNet and EfficientNetB0 respectively (AUC: 0.893 and 0.907). Meanwhile, 448 REZ achieve the best performance in Xception, DenseNet121 and ResNet50 (AUC: 0.900, 0.883 and 0.871 respectively). In physician-AI test set, the 320 REZ for EfficientNetB0 (AUC: 0.896, *P* < 0.1) is better than senior physicians. Besides, the 224 REZ for MobileNet (AUC: 0.878, *P* < 0.1), 448 REZ for Xception (AUC: 0.895, *P* < 0.1) are better than junior physicians. While the 448 REZ for DenseNet121 (AUC: 0.880, *P* < 0.05) and ResNet50 (AUC: 0.838, *P* < 0.05) are only better than entry physicians.

**Conclusion:**

Based on the gray-scale ultrasound breast images, we obtained the best DL combination which was better than the physicians.

## Highlights

1. Different combinations [model_resolution (REZ)] will yield different performance for the 2D grayscale breast ultrasound image classification, with Xception_448, MobileNet_224, EfficientNetB0_320, ResNet50_448, and DenseNet121_448 being the best choices.
2. MobileNet_224, EfficientNetB0_320 and Xception_448 can achieve equivalent performance as senior physicians.
3. The lightweight model, such as MobileNet_224, is more dominant in small REZ images, which also indicates that it is suitable for mobile application scenarios.
4. Image REZ has a slight effect on time consuming for model prediction, with a slight increase in time consuming for large REZ.

## Introduction

Breast cancer is the leading cause of death among women worldwide, with the highest incidence and the second highest mortality rate (1). Detecting and intervening in early stage could significantly improve 5-year-survival rate (2, 3).

Due to its portability and affordability, ultrasound (US) is most practical screening modality in different types of breasts, especially in dense breast (4). Since the fact that US is more accessible than mammography (5, 6), it is the first choice for breast early screening. However, the US is not sensitive enough to detect calcifications (7) and non-mass breast lesions (8, 9). Diagnose performance of US is highly operator-dependent. Therefore, there is an urgent need to find a method that is less operator-dependent and objectively reflects the nature of the tumor for breast cancer.

Deep learning (DL) can extract a large number of quantitative features from medical images, including features that are invisible to human eyes but could greatly improve diagnose accuracy (10–13). Breast US Artificial Intelligence (AI) can accurately identify breast masses in relation to the volume of masses (14–16). In addition, it can improve the diagnosis of early breast cancer, providing a reference for early diagnosis of non-mass lesions (17), determining the molecular subtypes (18, 19), pathological types (20), status of axillary lymph node metastasis (21, 22) and prognosis (23, 24). However, by analyzing and summarizing the current studies on intelligent discrimination based on breast US data, relationship of model_REZ and diagnose performance is not deeply explored. Lacking systematic review of model and parameters selection, no comparison between lightweight models (e.g., MobileNet, Xception, EfficientNetB0, etc.) (25–27) and heavyweight models (e.g., such as DenseNet121、ResNet50, etc.) (28, 29), no analysis and comparison of models under different image input REZs, and lack of comparison with physician diagnosis results. Therefore, the accuracy and precision of breast cancer diagnostic models and image combinations can be statistically determined by cross-comparison of multiple DL models and multiple image input REZs. Different models have different depth of networks, and the network depths are related to the input resolution. If we want to guarantee the best results of DL models, we should study the best combination of model depth and input REZ to ensure that a reasonably optimal models is used (30, 31).

Therefore, this study performed extensive cross-comparison of model_REZ and generalization tests: 1. Lightweight models (MobileNet, Xception and EfficientNetB0) and heavyweight models (DensNet121, and ResNet50). 2. Three dominant REZs (224 × 224 pixels, 320 × 320 pixels and 448×448 pixels). The above scientific hypotheses were verified by cross-comparisons: the diagnostic accuracy of the AI combinations (model_REZ) based on breast images is higher than senior physicians.

## Materials and Methods

### Research Objects

This multicenter study retrospectively examined 2D grayscale US breast images recruited from 2 Chinese hospitals from July 2015 to December 2020 with appropriate approval from the respective ethics committees. All benign and malignant nodules were confirmed pathologically after US testing.

Inclusion criteria: a. US-detected breast nodules, which diameter between 5.0 and 30.0 mm. b. Ability to show at least 3.0 mm of breast tissue around the nodule. c. Nodules must be Breast Imaging Reporting and Data System (BI-RADS) 0, 2, 3, 4a, 4b, 4c or 5. d. The nodules have not undergone interventional or surgery prior to the US examination. e. The patient underwent surgery or biopsy within 1 week of US data collection and pathology results were obtained.

Exclusion criteria: a. Normal breast. b. History of breast surgery or intervention. c. Poor image quality. d. without pathological results.

### Instruments

The Philips, GE, and Mindray equipment were chosen to increase the model’s adaptability. The image sources are spread randomly and uniformly. The details of the instrument are listed below.

- LOGIQ E9 (GE Medical Systems Ultrasound and Primary Care Diagnostics, USA) with ML6-15-D linear array probe.
- EPIQ 5 (Philips Ultrasound, Inc. USA) with L12-5 linear array probe.
- Resonan 7 (Mindray, China) with L11-3U linear array probe.

### Data Preparation

**Figure 1:**
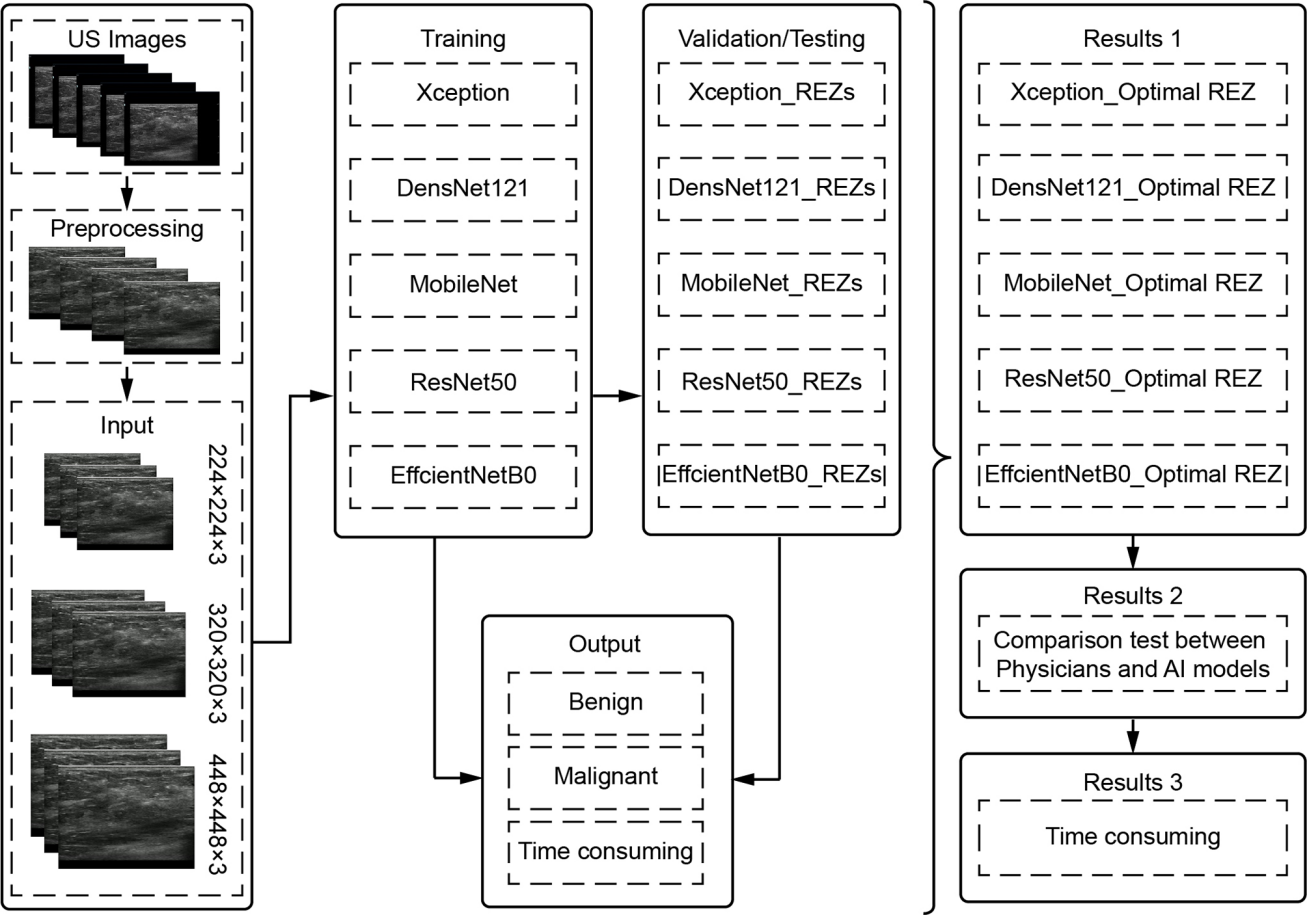
Architecture diagram. US, ultrasound; REZ, resolution.

To filter the combinations (model REZ) suited for breast cancer US picture classification, three REZs (224×224、320×320 and 448×448) and five deep learning models (Xception, DenseNet121, MobileNet, ResNet50, and EfficientNetB0) were trained and tested (**Figure 1**).

### Image Preprocessing

#### Image Cropping

The field-of-view (FOV) is extracted from the original image using image processing techniques by cropping out the device and patient-related information and keeping only the image window. Each FOV images is filled into a square and scaled to 224 × 224, 320 × 320, and 448 × 448, used as input data for model training and testing.

#### Image Enhancement

Prior to training the model, all image enhancements were performed using Python 3.9 as an automatic pre-processing process. This included Gaussian noise probability: 0.3, left-right flip probability: 0.5, rotation angle: -0.1: 0.1, X-axis shift: -0.1: 0.1, Y-axis shift: -0.1: 0.1, scaling. 0.7: 1, gamma correction. 0.6 to 1.6, stretching (left-right 50px, down 100px) and non-local average denoising (filter strength = 3, template window size = 7, search window size = 21).

### Model Training and Validation

The data were divided into training, validation and test set in a ratio of 8:1:1. Also, it was ensured that all images of the same patient appeared in the same set.

The default setting of training 100 epochs, while setting EarlyStop, 15 epochs of validation set loss does not drop will end the training early. The default setting of batch_size is (32).

### Model Testing

Internal and external tests were included in the test set. Three hundred images were randomly chosen from the test set for a comparison test of physicians and AI models, with the goal of determining not only the clinical usefulness of the models, but also whether the models’ diagnostic capabilities exceed those of physicians. The physcians did not known the results of AI and pathology. The process is as follows:

- The AI combinations (model_REZ) make diagnoses independently.
- Two physicians each from entry (<2 years), junior (2-5 years), and senior (>5 years) levels performed the diagnosis based on BI-RADS (33), which includes features such as size, shape, orientation, margin, echo pattern, posterior features, calcification and associated features.

### Statistical Analysis

Python 3.9 was applied for statistical analysis. The significance level was set at *P* = 0.05. In physician-model test, *P* = 0.1 was considered statistically significant.

The Kolmogorov-Smirnov test was used as a normality test. If the normal distribution was matched, variables were expressed as mean ± standard deviation (SD). If not, the median and interquartile range (IQR) are reported. The values of categorical variables are expressed as number (%). Within-group differences were compared using the paired-samples to test for continuous variables conforming to a normal distribution, and the Mann-Whitney U test for non-normal continuous variables. The Wilcoxon test was used to compare the error between physicians and AI combinations. The Kappa test was used to determine the intra-group consistency among different levels of physicians. The Specificity, sensitivity, accuracy, receiver operating characteristic curve (ROC), area under the curve (AUC) and 95% confidence interval (95% CI) were used for evaluation.

## Result

**Figure 2:**
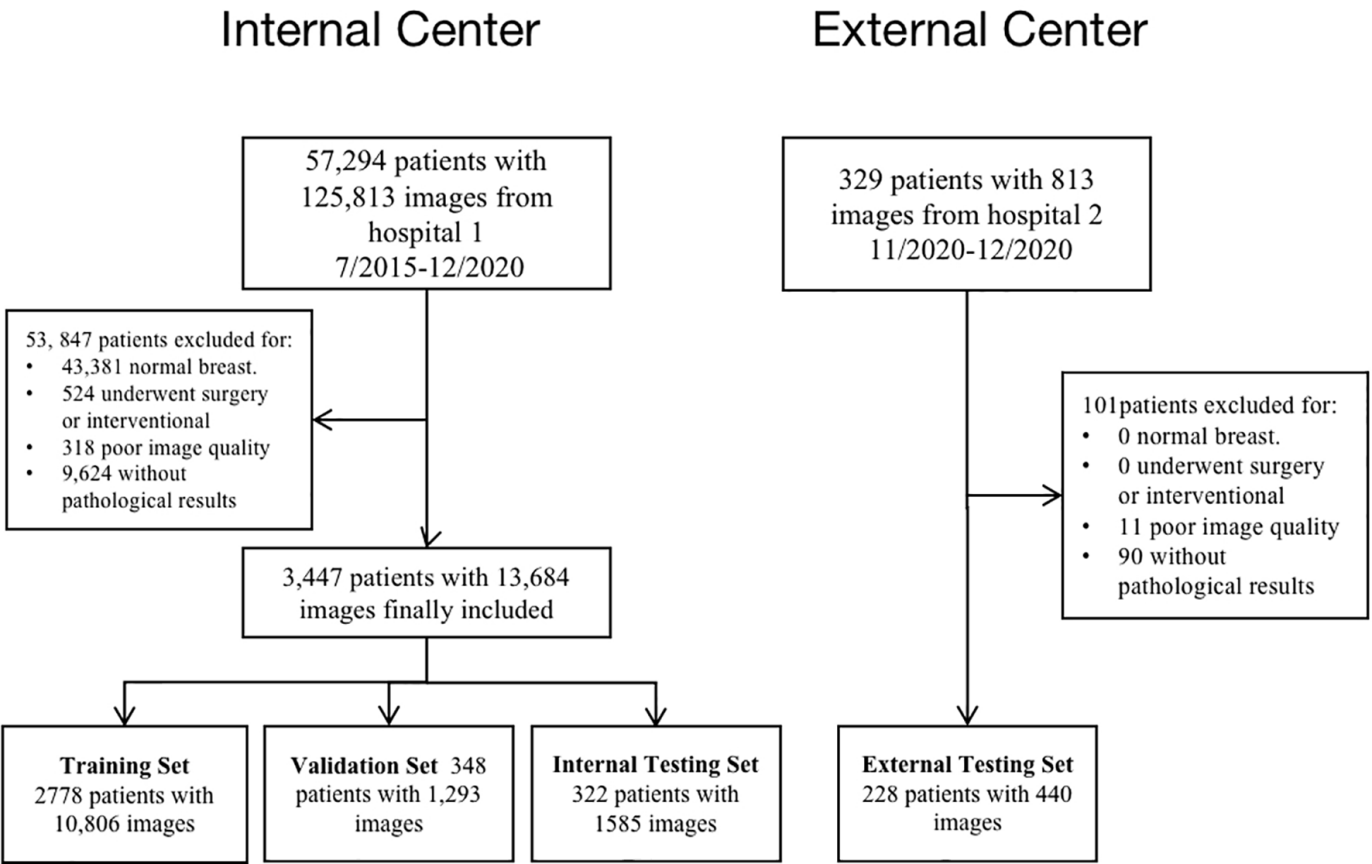
Study flow chart depicting patient enrollment at two hospitals.

A final total of 13,684 grayscale US images from 3,447 female patients were included according to the inclusion and exclusion criteria from July 2015 to December 2020. On average, 4 valid grayscale US images were available for each patient. Of these, 2457 were benign tumors (9102 images) and 990 were malignant tumors (4582 images). The training set, validation set, and internal test set are 10,806, 1,293, and 1,585 images, respectively, according to the 8:1:1 of the number of patients. The external testing set are 440 images respectively. **Table 1** show the distribution of baseline characteristics of the collected patients. **Appendix Tables 1**, **2** demonstrate the distribution of the study sample across trials. The flow chart is shown in **Figure 2**.

**Table 1:** Distribution of baseline characteristics of patients.

Variables	Benign (n=2457)	Malignant (n=990)	*P*
Age, year, mean ± SD	42.0 ± 11.7	46.0 ± 10.5	<0.001
Size, mm, mean ± SD	18 ± 6.9	21 ± 8.7	<0.001
Pathology, n			
Fibroadenoma	1161	--	
Adenosis of Breast	501	--	
Intraductal Papilloma	92	--	
Other Benign Tumors	703	--	
Infiltrative Non-specific Type of Carcinoma	--	560	
Ductal Carcinoma *in Situ*	--	49	
Infiltrating ductal carcinoma	--	17	
Infiltrating lobular carcinoma	--	21	
Other malignant tumors	--	343	

SD, standard deviation.

Parametric continuous variables are represented by mean ± SD and non-parametric variables are represented by median (IQR).

### Diagnosis of AI Models

#### Internal Test

For MobileNet, 448 REZ achieve the best AUC (0.886), with sensitivity 80.59%, specificity 80.97% and accuracy 80.59%. For Xception, 224 REZ achieve the best AUC (0.896), with sensitivity 77.31%, specificity 87.74, accuracy 83.53%. For EfficientNetB0, 320 REZ achieve the best AUC (0.887), with sensitivity 80.28%, specificity 83.30% and accuracy 82.08%. For DenseNet121, 224 REZ achieve the best AUC (0.867), with sensitivity 77.46%, specificity 81.40% and accuracy 79.81%. For ResNet50, 448 REZ achieve the best AUC (0.851), with sensitivity 72.61%, specificity 82.88% and accuracy 78.74%. The detailed results were summarized in **Table 2**.

**Table 2:** The results of all combinations (Model_REZ) in internal test set.

Models	AUC (95%CI)	Sen (%)	Spe (%)	Acc (%)
Xception				
224×224	0.896 (0.880-0.913)	77.31	87.74	83.53
320×320	0.883 (0.866-0.899)	81.22	81.08	81.14
448×448	0.887 (0.869-0.904)	83.41	82.14	82.65
MobileNet				
224×224	0.877 (0.859-0.895)	80.75	81.40	81.14
320×320	0.867 (0.849-0.886)	81.38	78.54	79.68
448×448	0.886 (0.870-0.903)	80.59	80.97	80.59
EfficientNetB0				
224×224	0.878 (0.861-0.895)	79.34	81.92	80.88
320×320	0.887 (0.870-0.904)	80.28	83.30	82.08
448×448	0.875 (0.857-0.893)	74.49	89.01	83.15
ResNet50				
224×224	0.781 (0.758-0.804)	72.46	70.08	71.04
320×320	0.847 (0.827-0.866)	77.93	76.96	77.35
448×448	0.851 (0.832-0.870)	72.61	82.88	78.74
DenseNet121				
224×224	0.867 (0.849-0.885)	77.46	81.40	79.81
320×320	0.849 (0.829-0.869)	79.34	78.54	78.86
448×448	0.866 (0.848-0.884)	79.03	80.97	80.19

REZ, resolution; AUC, area under the curve; CI, confidence interval; Sen, sensitivity; Spe, specificity; Acc, accuracy.

#### External Test

For MobileNet, 224 REZ achieve the best AUC (0.893), with sensitivity 81.59%, specificity 82.09% and accuracy 81.82%. For Xception, 448 REZ achieve the best AUC (0.900), with sensitivity 79.92%, specificity 88.56% and accuracy 83.86%. For EfficientNetB0, 320 REZ achieve the best AUC (0.907), with sensitivity 91.63%, specificity 72.14% and accuracy 82.73%. For DenseNet121, 448 REZ achieve the best AUC (0.883), with sensitivity 66.95%, specificity 94.53% and accuracy 79.55%. For ResNet50, 448 REZ achieve the best AUC (0.871), with sensitivity 79.92%, specificity 81.09% and accuracy 80.45%. The statistical results in detail are outlined in **Table 3**.

**Table 3:** The results of all combinations (Model_REZ) in external test set.

Models	AUC (95%CI)	Sen (%)	Spe (%)	Acc (%)
Xception				
224×224	0.885 (0.855-0.915)	74.48	87.56	74.48
320×320	0.832 (0.795-0.869)	69.46	82.59	75.45
448×448	0.900 (0.872-0.928)	79.92	88.56	83.86
MobileNet				
224×224	0.893 (0.864-0.922)	81.59	82.09	81.82
320×320	0.869 (0.836-0.902)	76.99	82.59	79.55
448×448	0.871 (0.839-0.903)	64.44	94.03	77.59
EfficientNetB0				
224×224	0.869 (0.836-0.901)	75.31	84.58	79.55
320×320	0.907 (0.880-0.934)	91.63	72.14	82.73
448×448	0.874 (0.842-0.906)	81.17	80.60	80.91
ResNet50				
224×224	0.788 (0.747-0.830)	68.20	77.11	72.27
320×320	0.838 (0.801-0.875)	75.31	80.60	77.73
448×448	0.871 (0.838-0.904)	79.92	81.09	80.45
DenseNet121				
224×224	0.801 (0.759-0.842)	58.16	92.54	73.86
320×320	0.848 (0.812-0.883)	75.31	80.60	77.73
448×448	0.883 (0.852-0.913)	66.95	94.53	79.55

REZ, resolution; AUC, area under the curve; CI, confidence interval; Sen, sensitivity; Spe, specificity; Acc, accuracy.

### Physician-AI Test Set

The consistency and accuracy of the diagnosis improved with the clinical experience of the physicians. The above results are shown in Appendix Table 3.

**Figure 3:**
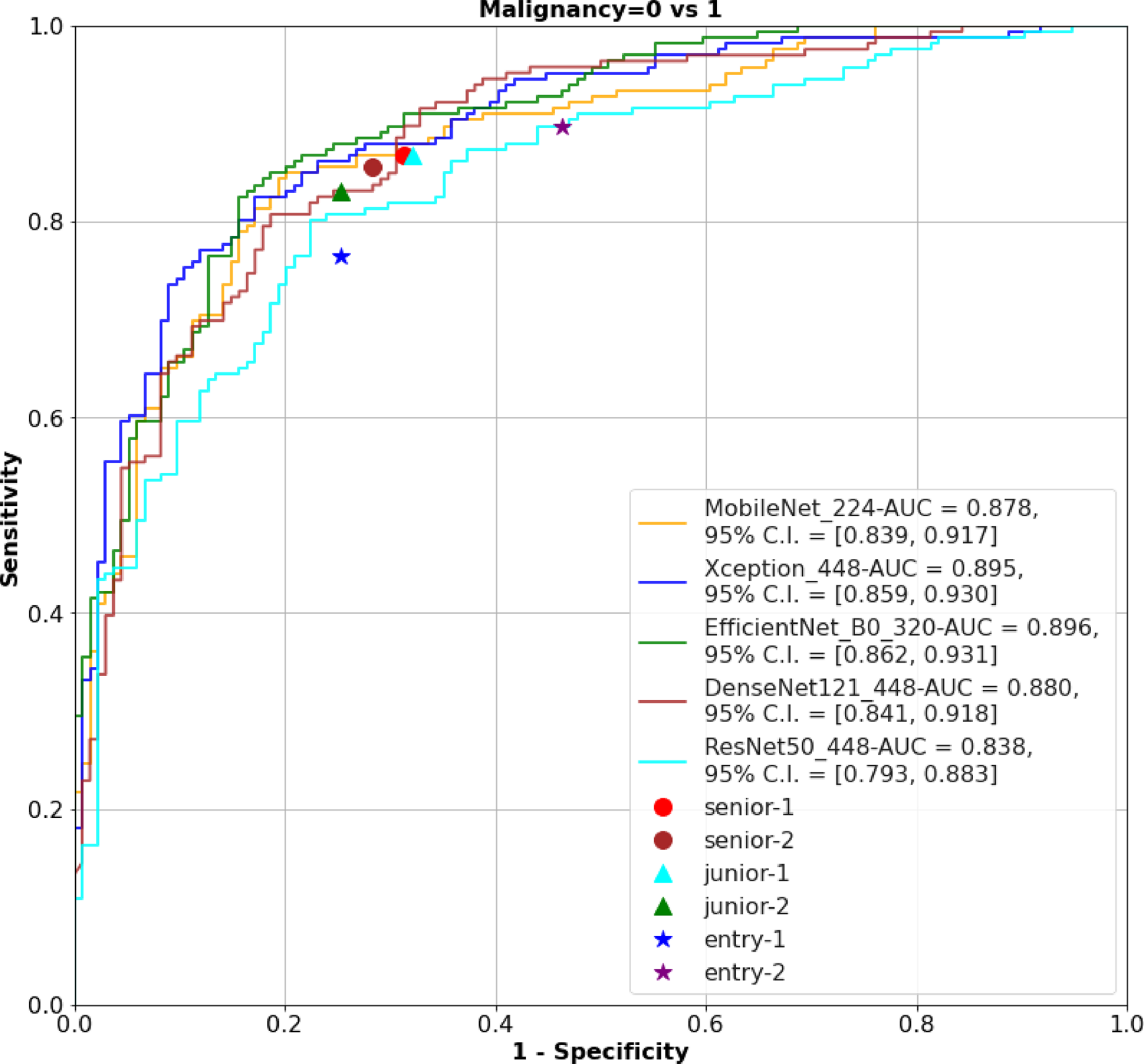
The ROC of the AI models and physicians. ROC, receiver operating characteristic; AI, Artificial Intelligence; AUC, area under curve.

**Figure 4:**
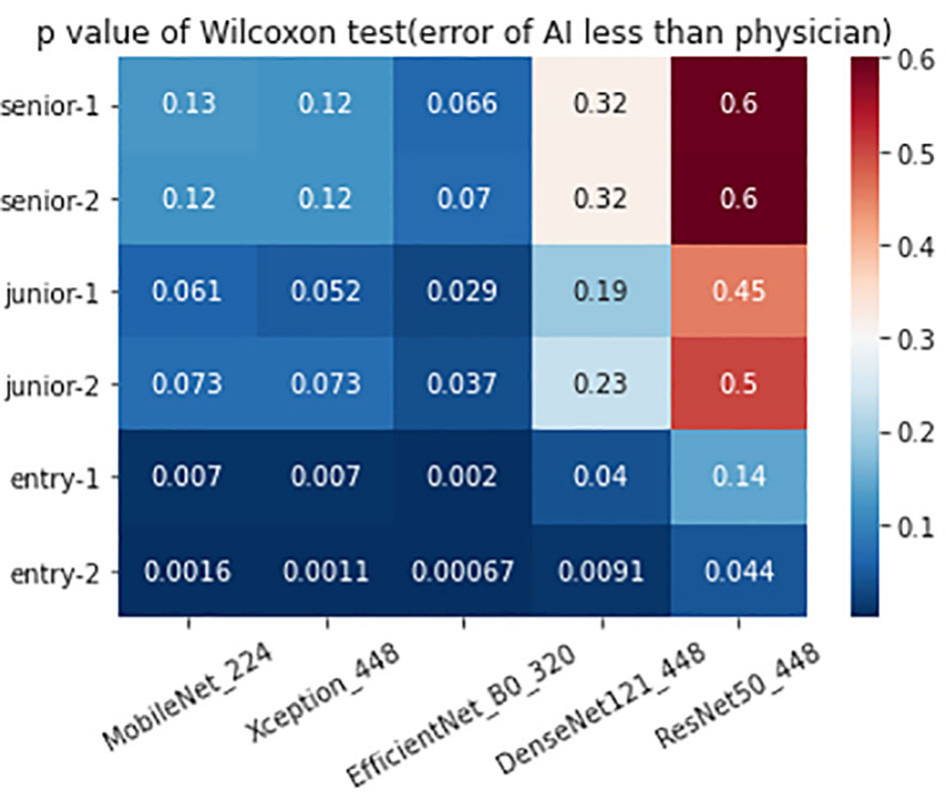
The heat map showed the p value of the physician-AI test set.

The 320 REZ for EfficientNetB0 (AUC: 0.896, *P* < 0.1) is better than senior physicians. The 224 REZ for MobileNet (AUC: 0.878, *P* < 0.1), 448 REZ for Xception (AUC: 0.895, *P* < 0.1) are better than junior physicians. The 448 REZ for DenseNet121 (AUC: 0.880, *P* < 0.05) and ResNet50 (AUC: 0.838, *P* < 0.05) are only better than entry physicians. The more detailed findings are presented in **Figures 3**, **4**.

### Time Consuming

#### Time Consuming to Training Models

Each Epoch took 11.06 ± 1.26 min (*P* < 0.0001); each Batch required 1.97 ± 0.22 s (*P* < 0.0001), and theoretical training of 50 Epochs took 9.22 ± 1.05 h (*P* < 0.0001), with statistically significant variations (**Appendix Table 4**).

#### The Average Time of Predicting an Image

**Figure 5:**
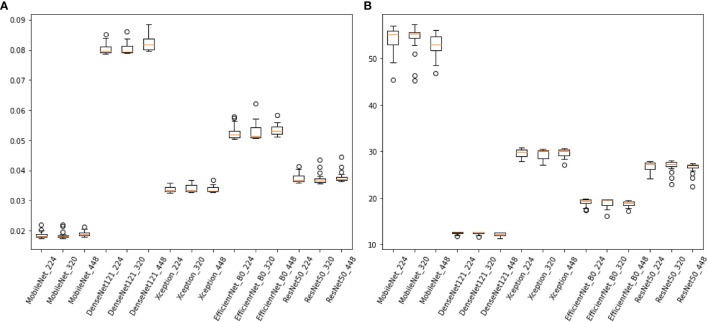
**(A)**, the consuming time of predicting an image of models (sec/frame). **(B)** the frame rate of models (sec/frame).

The difference in the average time taken by different models to predict an image is statistically significant. MobileNet_224 and 320 are the fastest models, both predicting within 0.02 s. The frame rate can reach 50 Frame/s, which is almost 1/4-1/3 of the Densenet121 time (**Figure 5**). REZ had an effect on the elapsed time of the model. A slight increase in elapsed time was observed for high REZ model predictions, with statistically significant differences within groups (**Table 4**; **Figure 5**).

**Table 4:** The average time (s) of predicting an image.

Models	REZ			
	224×224	320×320	448×448	*P*
DenseNet121	0.0753	0.0756	0.0772	<0.0001
MobileNet	0.0192	0.0199	0.0205	0.0004
Xception	0.0310	0.0337	0.0345	0.001
EfficientNetB0	0.0473	0.0476	0.0476	<0.0001
ResNet50	0.0342	0.0359	0.0373	0.0006
*P*	0.0124	<0.0105	<0.0102	

s, seconds; SD, standard deviation; REZ, resolution.

Parametric continuous variables are represented by mean ± SD and non-parametric variables are represented by median (IQR).

## Discussion

This study used 15 models to classify breast cancers at a higher degree than previous studies (32–35). The results showed that MobileNet_224, Xception_448 and EfficientNetB0_320 models showed the best diagnostic ability in tests set and physician-AI test set. This is the first study that we are aware of that specifically describes intra/intergroup comparisons of multiple models and REZs based on grayscale US breast images.

End-to-end philosophy of DL has benefited a lot for reducing the heavy workload preparing datasets. Only pathological findings were used as markers in this investigation, with no annotation of US pictures. It significantly reduces the initial workload. This study and a previous study (36) of our team found that, not only did this DL method produce good diagnostic results, but the diagnostic idea was similar to that of the physician.

The 224×224, 320×320 and 448×448 pixels were adopted in this study because these three REZs are commonly used in engineering (18, 22, 37). They are both obtained after scaling on the original size, and the different REZs react to the same model affecting the size of the convolutional kernel field of perception (38). In convolutions, images with small REZs do consume less computation time than large REZ, but may lose more information and produce misleading results (39). In this research, we found an increase in the consumption time of the model fed by high-REZ images, which is consistent with what has been reported in the literatures (40).

In both the external and physician-AI tests, MobileNet_224, EfficientNetB0_320, and Xception_448 surpass the other four models and all physicians. MobileNet can be a vision model built on handheld and mobile equipment. Although small image REZ has its own drawbacks (39), we can appropriately reduce the image REZ to increase speed to benefit portable US devices while having good accuracy and efficiency (25). While the high REZ of the input image (320×320) may increase computational cost, EfficientNetB0 uses an efficient convolutional neural networks architecture to process slightly larger images at a relatively similar cost to smaller images (41, 42). Using depth-segmented convolution in Xception provides similar properties to the original module, while being as easy to use as a normal convolutional layer. The convergence process is faster and more accurate when the nonlinear activation function is not used (43). Thus, when the input REZ is further increased to 448 × 448, Xception_448 is the best. This study found some difficulties in the selection of image inputs for ResNet50 and DenseNet121. In physician-AI test set, ResNet50_448 and DenseNet121_448 had only higher AUCs than those of entry physicians.

More data and richer data types are obvious ways to improve the efficiency of model diagnosis (44). The imbalance of the sample has an effect on the model. If all the data are trained together, it may appear balanced on the surface, but this can be masked in the real world. Sample imbalance at different aspects can have an impact on the final results of the model (45). Therefore, developing “one model for all situations” may still be a long process. Currently, more breast AI experiments of US with targeted and multimodal have been designed to address some problems on a smaller scale. They may become a more achievable goal in the short term, such as the prediction of lymph node metastasis, molecular subtypes and pathological types of breast cancer (18–22).

The hypothesis of this study is that AI is superior to physicians. And in order to reduce the type 2 errors in statistics and prevent the result that AI dominates the physician’s diagnosis, we argue that setting P to 0.1 is critical in the physician-AI test set. This is because AI is supposed to be an assistant not a decision maker that substitutes the doctor. If the type 2 error is too large, it will lead to more reliance on AI, which is a bad news for medicine, especially for hospitals in remote areas or less experienced physicians.

Numerous breast AI of US studies have demonstrated the amazing utility and reliability of this technique (14–24). But previous efforts have focused more on what AI can do and how far it can go, ignoring the differences in models across REZs, devices, and even users. Therefore, a study of these combinations (model_REZ) is necessary. It balances the efficiency, accuracy and reliability of breast AI of US, and provide a theoretical basis for specific equipment demands in future.

The data is often divided into training, validation and test sets following a ratio of 8:1:1 and 7:1:2 (46–48). When the sample size is small and the test set needs more, 7:1:2 is preferred. In this study, the sample size is 13,684, which is relatively large in ultrasound AI researches, thus we choose the ratio of 8:1:1. The sample size of the study by Ren was close to our study (47).

There are some limitations to this research. a: The study’s sample size was small, and the pathology was unevenly distributed. The origin of the instruments was not differentiated. Subsequently, more subgroups could be added for analysis. b: This study is a retrospective analysis with only a small amount of external validation data. c: Only static images were analyzed in this study. Video data and a multi-omics data can be added later to improve the richness of training.

## Conclusion

Based on unlabeled 2D grayscale images of breast US, this study obtained the optimal combinations (model_REZ) and outperformed the entry, junior and senior practitioners. This study also reveals the promising application of unlabeled ROI in medical imaging of DL, which greatly reduces the cost and time.

## Data Availability Statement

The data analyzed in this study is subject to the following licenses/restrictions: We have further studies that require this database. Requests to access these datasets should be directed to HW, 234721800@qq.com.

## Ethics Statement

The studies involving human participants were reviewed and approved by Shenzhen people’s hospital of the ethics committee. The patients/participants provided their written informed consent to participate in this study.

## Author Contributions

HW contributed to the study concepts, designed the study, HW and XY collected and organized all the images; YJ contributed to the model training, validation, testing, and statistical analysis. HW and YJ contributed to data analysis and interpreted the data and HW prepared the manuscript. They contributed to the study equally and shared the first authorship.

## Funding

This project was supported by Commission of Science and Technology of Shenzhen (GJHZ20200731095401004).

## Conflict of Interest

Authors YJ, CC and SS were employed by Microport Prophecy and Illuminate Limited Liability Company.

The remaining authors declare that the research was conducted in the absence of any commercial or financial relationships that could be construed as a potential conflict of interest.

## Publisher’s Note

All claims expressed in this article are solely those of the authors and do not necessarily represent those of their affiliated organizations, or those of the publisher, the editors and the reviewers. Any product that may be evaluated in this article, or claim that may be made by its manufacturer, is not guaranteed or endorsed by the publisher.

## Abbreviations

AI, Artificial Intelligence; AUC, area under curve; BI-RADS, breast imaging reporting and data system; CI, confidence interval; DL, Deep Learning; IQR, interquartile range; ML, Machine Learning; ROC, receiver operating characteristic; ROI, region of interest; SD, standard deviation; SL, supervised learning; SSL, semi-supervised learning; US, Ultrasound.
