# Supplementary material for: A Comparative Study of Multiple Deep Learning Models Based on Multi-Input Resolution for Breast Ultrasound Images
Source: Front Oncol. 2022 Jul 7;12:869421. doi: 10.3389/fonc.2022.869421 (PMC9302001; doi:10.3389/fonc.2022.869421)
Supplement: Supplementary file 1 [file Table_1.docx]

**Appendix Table 1. The distribution of the study sample in training, validation and testing sets**

|  | BIRADS | 0 | | 2 | | 3 | | 4a | | 4b | | 4c | | 5 | | Total |
| --- | --- | --- | --- | --- | --- | --- | --- | --- | --- | --- | --- | --- | --- | --- | --- | --- |
|  |  | B | M | B | M | B | M | B | M | B | M | B | M | B | M |  |
| **Training Set** | Patients, n, % | 9 | 2 | 27 | 2 | 1309 | 32 | 554 | 78 | 87 | 183 | 17 | 419 | 1 | 58 | 2778 |
|  |  | 82% | 18% | 93% | 7% | 98% | 2% | 88% | 12% | 32% | 68% | 4% | 96% | 2% | 98% |  |
|  | Images, n, % | 25 | 4 | 60 | 4 | 4380 | 69 | 2411 | 327 | 382 | 779 | 63 | 1989 | 3 | 310 | 10806 |
|  |  | 86% | 14% | 94% | 43% | 98% | 2% | 88% | 12% | 33% | 67% | 3% | 97% | 1% | 99% |  |
| **Validation Set** | Patients, n, % | 1 | 0 | 4 | 1 | 167 | 4 | 69 | 9 | 12 | 24 | 1 | 48 | 0 | 8 | 348 |
|  |  | 100% | 0% | 80% | 20% | 98% | 2% | 88% | 12% | 33% | 67% | 2% | 98% | 0% | 100% |  |
|  | Images, n, % | 2 | 0 | 11 | 1 | 548 | 7 | 266 | 25 | 56 | 100 | 6 | 235 | 0 | 36 | 1293 |
|  |  | 100% | 0% | 92% | 8% | 99% | 1% | 91% | 9% | 36% | 64% | 2% | 98% | 0% | 100% |  |
| **Internal Testing Set** | Patients, n, % | 1 | 0 | 1 | 1 | 136 | 3 | 56 | 13 | 5 | 22 | X120 | 55 | 0 | 29 | 322 |
|  |  | 100% | 0% | 50% | 50% | 99% | 2% | 81% | 19% | 19% | 81% | 0% | 100% | 0% | 100% |  |
|  | Images, n, % | 3 | 0 | 2 | 1 | 613 | 9 | 306 | 47 | 22 | 116 | 0 | 322 | 0 | 144 | 1585 |
|  |  | 100% | 0% | 67% | 33% | 99% | 1% | 87% | 13% | 16% | 84% | 0% | 100% | 0% | 100% |  |
| **External Testing Set** | Patients, n, % | 0 | 0 | 1 | 0 | 130 | 4 | 24 | 18 | 2 | 15 | 0 | 15 | 0 | 19 | 228 |
|  |  | 0% | 0% | 100% | 0% | 97% | 3% | 57% | 43% | 12% | 88% | 0% | 100% | 0% | 100% |  |
|  | Images, n, % | 0 | 0 | 1 | 0 | 170 | 10 | 28 | 64 | 2 | 40 | 0 | 43 | 0 | 82 | 440 |
|  |  | 0% | 0% | 100% | 0% | 94% | 6% | 30% | 70% | 5% | 95% | 0% | 100% | 0% | 100% |  |

Abbreviations: B, benign; M, malignant

**Appendix Table 2. The distribution of the study sample in physicians-AI test set**

| BIRADS | 3 | | 4a | | 4b | | 4c | | 5 | | Total |
| --- | --- | --- | --- | --- | --- | --- | --- | --- | --- | --- | --- |
|  | B | M | B | M | B | M | B | M | B | M |  |
| Patients, n, % | 92 | 2 | 33 | 8 | 1 | 14 | 1 | 34 | 0 | 35 | 220 |
|  | 98% | 2% | 80% | 20% | 7% | 93% | 3% | 97% | 0% | 100% |  |
| Images, n, % | 98 | 2 | 34 | 9 | 1 | 16 | 1 | 39 | 0 | 100 | 300 |
|  | 98% | 2% | 79% | 21% | 6% | 94% | 3% | 98% | 0% | 100% |  |

Abbreviations: B, benign; M, malignant

**Appendix Table 3. The results of physicians diagnosis**

| Physicians | Sen (%) | Spe (%) | Acc (%) | Kappa | *P* |
| --- | --- | --- | --- | --- | --- |
| Entry 1 | 76.51 | 74.63 | 75.67 | 0.533 | 0.00 |
| Entry 2 | 89.76 | 53.73 | 73.67 |  |  |
| Junior 1 | 86.75 | 68.66 | 78.67 | 0.620 | 0.043 |
| Junior 2 | 86.75 | 67.91 | 78.33 |  |  |
| Senior 1 | 83.13 | 74.63 | 79.33 | 0.608 | 0.424 |
| Senior 2 | 85.54 | 71.64 | 79.33 |  |  |

Abbreviations: Sen, sensitivity; Spe, specificity; Acc, accuracy; SD, standard deviation.

**Appendix Table 4. Time consumption for training different models**

| Models | Time per Epoch (min) | Time per Batch (s) | Estimated training time (h) |
| --- | --- | --- | --- |
| **MobileNet** | 11.92 | 2.12 | 9.93 |
| **Xception** | 9.56 | 1.70 | 7.97 |
| **EfficientNetB0** | 11.89 | 2.12 | 9.91 |
| **DenseNet121** | 12.10 | 2.15 | 10.1 |
| **ResNet50** | 9.81 | 1.75 | 8.18 |
| **Mean ± SD** | 11.06 ± 1.26 | 1.97 ± 0.22 | 9.22 ± 1.05 |
| ***P*** | ＜0.0001 | ＜0.0001 | ＜0.0001 |

Abbreviations: min, minutes; s, seconds; h, hours; SD, standard deviation.

Note: The estimated training time is based on the assumption of training on 50 Epochs. Parametric continuous variables are represented by mean ± SD and non-parametric variables are represented by median (IQR)
